# Prevalence and correlates of suicide attempts in Chinese individuals with borderline personality disorder

**DOI:** 10.3389/fpsyt.2022.942782

**Published:** 2022-08-29

**Authors:** Fan Yang, Jun Tong, Shu-Fang Zhang, Juan Zhang, Bao-Liang Zhong

**Affiliations:** ^1^Department of Psychiatry, Wuhan Mental Health Center, Wuhan, China; ^2^Department of Psychiatry, Wuhan Hospital for Psychotherapy, Wuhan, China

**Keywords:** borderline personality disorder, suicide attempt, prevalence, factors, clinical characteristics

## Abstract

**Background:**

To date, few empirical studies have examined the clinical characteristics of suicide attempts (SA) in individuals with borderline personality disorder (BPD) in China.

**Aims:**

To examine the prevalence and factors associated with SA in Chinese individuals with BPD.

**Methods:**

In this cross-sectional study, 84 patients with BPD were recruited from a large public psychiatric hospital in Wuhan, China, between 2013 and 2015. Trained experienced psychiatrists interviewed participants to collect clinical data, including demographics, axis I and axis II diagnoses of mental disorders according to the DSM-IV-TR, number of hospitalizations, and history of SA. An interview outline was used to identify the existence of lifetime SA. In addition, the Beck Depression Inventory-II, Buss & Perry Aggression Questionnaire, Child Trauma Questionnaire-Short Form, and Beck Hopelessness Scale were administered to assess respondents’ depressive symptoms, aggression, childhood traumatic experiences, and hopelessness.

**Results:**

Fifty-two (61.9%) patients reported attempting suicide during their lifetime. Univariate logistic regression analysis screened 7 factors associated with SA in individuals with BPD into Multiple logistic regression analysis: female sex, unemployment, major depressive disorder (MDD), hostility, self-aggression, depressive symptoms, and emotional neglect. Multiple logistic regression analysis identified 3 significant and independent correlates of SA: MDD [odds ratio (OR) = 26.773, 95% confidence interval (CI) = 3.914–183.132, *P* = 0.001], hostility (OR = 1.073, CI = 1.019–1.130, *P* = 0.007), and self-aggression (OR = 1.056, CI = 0.998–1.119, *P* = 0.060).

**Conclusion:**

Chinese individuals with BPD have a high risk of suicide. Correlates of SA in this population differ to some extent from those in Western populations as reported in the literature. Paying attention to MDD and some types of aggression in Chinese individuals with BPD may help identify their risk of suicide. Future large-sample cohort study may improve the limitations of this study and further confirm the point of view above.

## Introduction

The main characteristics of borderline personality disorder (BPD) include a pervasive pattern of emotion dysregulation, inconsistent identity, and disturbed interpersonal function, as indicated by five (or more) of the following: fear of abandonment, unstable relationships, unstable self-image, impulsivity, recurrent suicide/self-harm, mood instability, feelings of emptiness, inappropriate anger, dissociation/transient paranoid ideation (1). BPD is the most common personality disorder in both clinical and non-clinical settings in Western countries, with a prevalence of approximately 10–25% (2, 3) and 0.7–5.9% (4–6) in clinical and non-clinical populations, respectively. In China, a multicenter study reported that the prevalence of BPD reached 9.9% in psychiatric treatment settings (7). BPD is a serious personality disorder that can cause severe social function impairments, and it is associated with a high risk of suicide. It has been documented that approximately 50–75% of patients with BPD have attempted suicide (8, 9); in addition, each of these patients has attempted suicide more than three times during their lifetime (10). Repetitive suicidal behaviors in patients with BPD are sometimes misinterpreted as merely a form of threat or manipulation in clinical practice. However, since suicide attempts (SA) lead to a greater risk of completed suicide than expected (11), it is a significant clinical concern in patients with BPD.

Several factors were probably associated with SA in patients with BPD, such as comorbidities of other mental disorders, personality traits, childhood abuse, some demographic characteristics, and other factors; however, studies have shown inconsistent results to some extent (10, 12–20). Many studies on BPD indicated that there were significant associations between SAs and affective disorders, especially depressive disorders (10, 14, 17). However, few found that a comorbid diagnosis of major depression had only a short-term effect or little effect on suicide risk in patients with BPD (18, 19). One study even suggested further that risk factors for SA include state depression (e.g., depressed symptoms), but not comorbid affective disorder (18). Similarly, substance abuse is common among suicide attempters with BPD (13, 14); however, some studies have found no associations between substance abuse and SA in these patients (18, 20). Another possible associated factor is the personality traits of patients with BPD, such as aggression, anger, hostility, and impulsivity (10, 12, 13, 15, 17, 19); but negative results have also been reported (20). Moreover, childhood abuse, especially sexual abuse, was considered correlated with SA in patients with BPD (15, 16). Low socioeconomic status, age, hospitalization, and poor psychosocial functioning have also been classified as risk factors for SA in patients with BPD (16, 19, 20).

As mentioned above, the data for clinical characteristics of suicidal behaviors in individuals with BPD are mainly derived from Western countries, and there are still some inconsistent findings. In China, to the best of our knowledge, few empirical studies have been conducted to examine the clinical epidemiology of SA in patients with BPD in China. From the few research data in China (7), it can be inferred that the number of Chinese patients in with BPD may be huge, and the suicidal behavior of this population is a clinical problem that cannot be ignored. Considering the clinical importance of suicide prevention and management of suicidal risk in patients with BPD, we investigated the prevalence and correlates of SA in patients with BPD from a large public psychiatric hospital in central China.

## Materials and methods

### Participants

Participants were selected from patients treated at Wuhan Mental Health Center over a period of 2 years from 2013 to 2015. This center is the largest public psychiatric specialty hospital in Wuhan, central China, with over 1,000 inpatient beds. Patients with confirmed clinical diagnosis of BPD, according to the Diagnostic and Statistical Manual of Mental Disorders, Fourth Edition, Text Revision (DSM-IV-TR) (21), aged between 18 and 60 years, and received treatments at the hospital during the study period were consecutively invited to join the study. Patients with severe physical diseases, including neurological diseases, such as epilepsy, were excluded. Two experienced psychiatrists assessed their eligibility and invited eligible patients to participate in the study.

### Assessment

Trained investigators interviewed patients in a standardized manner, aiming to collect demographic information (sex, age, educational background, occupation, and marital status), axis I diagnoses of DSM-IV-TR, number of hospitalizations, and the outcome of this study, lifetime SA. An interview outline was used to identify the existence of lifetime SA, which mainly included four questions: (1) Have you ever self-injured or suicided, such as taking medicine or cutting your wrists? (2) How many times? (3) What specific methods were used? (4) How much did you want to die while doing these things? If the patient answered yes to question 1 and was able to describe in detail at least one suicidal behavior with thoughts of death, SA was present; Otherwise, SA was absent.

All participants completed 4 validated Chinese self-rating scales: Buss and Perry Aggression Questionnaire (BP-AQ) (22), Beck Depression Inventory-II (BDI-II) (23), Child Trauma Questionnaire-Short Form (CTQ-SF) (24), and Beck Hopelessness Scale (BHS) (25). BP-AQ was used to assess personality characteristic of aggression. It consisted of 30 items, with five subscales: physical aggression, anger, verbal aggression, hostility, and self-aggression. The CTQ-SF was used to assess childhood abuse and consisted of 28 items in five subscales: emotional abuse, sexual abuse, physical abuse, physical neglect, and emotional neglect. BDI-II and BHS were used to rate the levels of depressive symptoms and hopelessness, respectively.

### Statistics

Descriptive statistics and univariate and multivariate logistic regression analyses were performed. In the univariate logistic regression analysis, the presence or absence of SA was considered as the outcome variable, while the above demographic, psychosocial, and clinical variables were considered as the independent variables. Significant factors identified in the univariate analysis were included into the multivariate logistic regression analysis. Forward stepwise regression based on maximum likelihood estimation was used to identify independent factors associated with SA in patients with BPD. The 95% confidence intervals (CIs) and odds ratios (ORs) were used to quantify the association between the factors and SA. The statistical significance level was set at two-sided *P* < 0.05. All analyses were performed using IBM SPSS 20.0 (IBM SPSS Statistics for Windows, Version 20.0. Armonk, NY: IBM Corp.).

## Results

### Characteristics of included patients

During the study period, a total of 355 patients with BPD met the inclusion and exclusion criteria, of which 84 patients agreed to participate in the study and completed the questionnaires, and all questionnaires were qualified for the analysis. Among the 84 patients with BPD, the age ranged from 18 to 38 years (mean age, 24.6 ± 5.5 years), and 52 (61.9%) had attempted suicide before the interview. Other clinical characteristics and demographic data are shown in Table 1.

**TABLE 1:** Characteristics of Chinese borderline personality disorder (BPD) patients (*N* = 84).

Characteristics	*N* (%)
**Gender**	
Male	22 (26.2)
Female	62 (73.8)
**Education level**	
Junior college below	32 (38.1)
Junior college or above	52 (61.9)
**Occupation**	
Unemployed	23 (27.4)
Student	33 (39.3)
Others	28 (33.3)
**Marital status**	
Unmarried	75 (89.3)
Married	5 (6.0)
Others (divorced, widowed or remarried)	4 (4.7)
**Number of hospitalizations**	
0 (outpatient)	5 (6.0)
1	10 (11.9)
2 or above	69 (82.1)
**DSM-IV-TR*^a^* axis I diagnosis**	
Major depressive disorder (MDD)	57 (67.9)
Bipolar disorder	8 (9.5)
Other or unspecified psychotic disorders	5 (6.0)
Post-traumatic stress disorder	4 (4.7)
No diagnosis	10 (11.9)

*^a^*Diagnostic and Statistical Manual of Mental Disorders, Fourth Edition, Text Revision.

### Results of the logistic regression analysis on the factors associated with suicide attempts in borderline personality disorder patients

The univariate logistic regression analysis (Table 2) revealed that there were 7 factors associated with SA in patients with BPD, namely female sex, unemployment, major depressive disorder (MDD), hostility, self-aggression, depressive symptoms, and emotional neglect.

**TABLE 2:** Results of the univariate logistic regression analysis on the factors correlated with suicide attempt (SA) in BPD patients.

Factors	B	S.E.	Wald χ ^2^	*P*	OR (95%CI)
Female (vs. male)	1.216	0.546	4.955	0.026	3.375 (1.156, 9.850)
Age	–0.019	0.044	0.174	0.677	0.982 (0.900, 1.071)
Junior college below (vs. Junior college or above)	–0.734	0.516	2.020	0.155	0.480 (0.174, 1.321)
Unemployed (vs. student and other occupations)	1.232	0.624	3.894	0.048	3.429 (1.008, 11.658)
Married (vs. other marital status)	–20.846	20096.485	0.000	0.999	0.000
Number of hospitalizations (<2 vs. ≥2)	–21.357	8987.429	0.000	0.998	0.000
MDD (vs. other axis I diagnosis)	3.219	0.671	23.025	<0.001	25 (6.713, 93.099)
Physical aggression	0.024	0.015	2.487	0.115	1.024 (0.994, 1.055)
Anger	0.022	0.012	3.504	0.061	1.022 (0.999, 1.046)
Verbal aggression	–0.001	0.012	0.012	0.912	0.999 (0.975, 1.023)
Hostility	0.055	0.017	10.356	0.001	1.057 (1.022, 1.093)
Self-aggression	0.081	0.021	15.404	<0.001	1.085 (1.041, 1.129)
Depressive symptoms	0.059	0.025	5.675	0.017	1.061 (1.011, 1.114)
Hopelessness	0.060	0.050	1.455	0.228	1.062 (0.963, 1.171)
Emotional abuse	0.098	0.060	2.647	0.104	1.103 (0.980, 1.242)
Sexual abuse	0.188	0.110	2.940	0.086	1.207 (0.973, 1.497)
Physical abuse	0.064	0.109	0.348	0.555	1.066 (0.862, 1.320)
Physical neglect	0.162	0.108	2.251	0.134	1.176 (0.952, 1.453)
Emotional neglect	0.317	0.101	9.842	0.002	1.373 (1.126, 1.674)

The final model of multivariate logistic regression analysis (Table 3) identified three correlates significantly and independently associated with SA in BPD patients: MDD (OR = 26.773, CI = 3.914–183.132, *P* = 0.001), hostility (OR = 1.073, CI = 1.019–1.130, *P* = 0.007), and self-aggression (OR = 1.056, CI = 0.998–1.119, *P* = 0.060).

**TABLE 3:** Results of the multivariate logistic regression analysis on the factors correlated with SA in BPD patients.

Factors	B	S.E.	Wald χ ^2^	*P*	OR (95%CI)
MDD (vs. other axis I diagnosis)	3.287	0.981	11.228	0.001	26.773 (3.914, 183.132)
Hostility	0.071	0.026	7.239	0.007	1.073 (1.019, 1.130)
Self-aggression	0.055	0.029	3.534	0.060	1.056 (0.998, 1.119)

## Discussion

To the best of our knowledge, this is the first study to examine SA and its associated factors in individuals with BPD in China. The results showed that the lifetime prevalence of SA in Chinese patients with BPD was 61.9%, which is consistent with the high rate reported in Western countries (8, 9). The significant correlates of SA in this patient population were MDD and self-aggression, partly replicating the findings from Western studies (10, 12–15, 17, 19). Meanwhile, it was preliminarily speculated from other inconsistent results that some of the differences might be related to different cultures and regions.

MDD, as a typical and severe type of depressive disorder, has been associated with SA in patients with BPD, which was also found in our study (10, 14, 17). Compared with depressive state and temporary psychological symptoms, MDD refers to more profound and persistent mood problems, and patients may eventually assume that suicide is the only way to end their painful lives. This accords with the characteristics of repeated SAs in patients with BPD. They tend not to present only occasionally suicidal impulses or depressive state. Understandably, the correlations of current depressive and hopeless symptoms to SA in this patient population were not found in this study, although previous studies had the opposite results (10, 18). In addition, no association between comorbidities of other mental disorders and SA in patients with BPD was found in this study, which may be partly due to the small number of cases with comorbidities of other mental disorders. Although this is inconsistent with some Western studies (13, 14), it supports the findings of a large sample study in Shanghai, another China city: depressive disorder is the most common axis I disorder in BPD clinical samples, while substance abuse is rare (26). The DSM-IV includes substance abuse in the diagnostic criteria for BPD as one of the self-damaging impulsive behaviors, but many drugs are tightly controlled in China, which greatly limits their availability.

Furthermore, aggression, especially hostility and self-aggression, are also significant correlates of SA in patients with BPD, partly replicating the findings of previous studies (10, 12, 13, 15, 17). From the psychopathology of BPD, the hate for important objects caused by severe psychological trauma in the early years and the symbiotic relationship with those objects leads to frequent no-boundary dissatisfaction and violence against others and oneself, and the extreme result is suicide. Specifically, most items of hostility involved the defense mechanism of projection, a paranoid belief that the outside world is bad, while refusing to acknowledge inner bad feelings. More importantly, self-aggression subscale was all about intentional or unintentional self-harm or self-punishment, which was conceptually related to SA. Therefore, this factor was still retained as a correlate of SA in this study, although its significance was borderline. In contrast, impulsiveness, another personality trait of BPD, was not included in this study on the basis of the possibility of overlap between aggression and impulsiveness (27, 28), and aggression might be more relevant to suicide in patients with BPD (10, 13).

The effect of childhood abuse was not shown in this study, and even sexual abuse, considered by some studies to be the strongest predictor of SA in patients with BPD (15, 16), was not found to be associated. Talking about sex is more of a taboo in Eastern cultures than in Western ones, and this is also true among people with mental disorders (29, 30), which may mask the role sexual abuse plays in such patients. However, why other types of abuse were not associated with SA requires further clarification. In addition, patients’ ages and number of hospitalizations were also not associated with SA in this study, which was not consistent with the results of some other BPD studies (16, 20). Most patients were young (under 38 years), which is in line with a significant decline in the prevalence of patients with BPD over 40 years (31), and most patients were repeatedly hospitalized, which is consistent with some studies (32, 33). This may be explained by repeated hospitalizations as a way to escape from daily life, while symptoms of the personality disorder tend to ease with age. Therefore, it may result in failure to distinguish differences in SA among patients of different ages and hospitalizations in this study.

This study had some limitations. First, the participants were clinical patients from a psychiatric hospital in a city in central China, and the sample size was relatively small, which suggested that the findings should be explained with considerable caution. Second, the cross-sectional nature of the study did not permit an examination of causality. Third, as this was a preliminary study, research tools require improving, and certain data require further collection and analysis, such as using a structured interview to improve the detection rate of BPD, comparing it with the results of a new dimension system in the diagnosis for personality disorders (DSM-5 or ICD-11), and investigating the family history of suicide, psychosocial functioning, frequency of SA and other factors. Future research, conducting a relatively large cohort study to compare suicide risk among patients at different time points, use improved diagnostic tools, collect more clinical variables, and collect patient data in community populations as controls, should address most of the limitations of this study.

## Conclusion

The high prevalence of SA in Chinese individuals with BPD suggests a significant risk of suicide in this population in China. The suicidal behavior of Chinese patients with BPD is more likely to accompany with MDD and personality characteristics of hostility and self-aggression, but is not related to substance abuse, childhood sexual abuse and other factors as reported by Western studies. Paying attention to MDD and some types of aggression in Chinese individuals with BPD may help identify their risk of suicide. However, given the small sample size, the results should be interpreted with caution. Future large-sample cohort studies incorporating community population sampling and optimizing research tools may further refine the limitations of this study.

## Data availability statement

The raw data supporting the conclusions of this article will be made available by the authors, without undue reservation.

## Ethics statement

The studies involving human participants were reviewed and approved by the Institutional Review Board of the Wuhan Health and Family Planning Commission [approval number (2013)9, 5/12/2013]. The patients/participants provided their written informed consent to participate in this study.

## Author contributions

FY and JT contributed to the conceptualization. JZ and S-FZ investigated patients and conducted project administration. B-LZ analyzed and interpreted the data. FY and S-FZ wrote the draft of the manuscript. B-LZ, JZ, and JT revised and edited the manuscript. FY acquired funding. B-LZ and JT provided supervision. All authors contributed to the article and approved the submitted version.
